# Achieving Good Outcomes for Asthma Living (GOAL): mixed methods feasibility and pilot cluster randomised controlled trial of a practical intervention for eliciting, setting and achieving goals for adults with asthma

**DOI:** 10.1186/s13063-016-1684-7

**Published:** 2016-12-08

**Authors:** Gaylor Hoskins, Brian Williams, Purva Abhyankar, Peter Donnan, Edward Duncan, Hilary Pinnock, Marjon van der Pol, Petra Rauchhaus, Anne Taylor, Aziz Sheikh

**Affiliations:** 1 Nursing, Midwifery and Allied Health Professions Research Unit, School of Health Sciences, University of Stirling, Unit 13 Scion House, Stirling University Innovation Park, Stirling, FK9 4NF Scotland, UK; 2 School of Nursing, Midwifery and Social Care, Edinburgh Napier University, 9 Sighthill Court, Edinburgh, EH11 4BN Scotland; 3 Faculty of Health Sciences and Sport, University of Stirling, Pathfoot Building, Stirling, FK9 4LA Scotland, UK; 4 Tayside Clinical Trials Unit, Level 10 Ninewells Hospital and Medical School, Dundee, DD1 9SY Scotland, UK; 5 Asthma UK Centre for Applied Research, Usher Institute of Population Health Sciences and Informatics, The University of Edinburgh, Medical School Doorway 3, Teviot Place, Edinburgh, EH8 9AG Scotland, UK; 6 Health Economics Research Unit, University of Aberdeen, Foresterhill, Aberdeen, AB25 2ZD Scotland, UK

**Keywords:** Asthma, Complex interventions, Goal setting, Mixed methods, Pilot cluster RCT, Self-management

## Abstract

**Background:**

Despite being a core component of self-management, goal setting is rarely used in routine care. We piloted a primary care, nurse-led intervention called Achieving Good Outcomes for Asthma Living (GOAL) for adults with asthma. Patients were invited to identify and prioritise their goals in preparation for discussing and negotiating an action/coping plan with the nurse at a routine asthma review.

**Methods:**

The 18-month mixed methods feasibility cluster pilot trial stratified and then randomised practices to deliver usual care (UC) or a goal-setting intervention (GOAL). Practice asthma nurses and adult patients with active asthma were invited to participate. The primary outcome was asthma-specific quality of life. Semi-structured interviews with a purposive patient sample (*n* = 14) and 10 participating nurses explored GOAL perception. The constructs of normalisation process theory (NPT) were used to analyse and interpret data.

**Results:**

Ten practices participated (five in each arm), exceeding our target of eight. However, only 48 patients (target 80) were recruited (18 in GOAL practices). At 6 months post-intervention, the difference in mean asthma-related quality of life (mAQLQ) between intervention and control was 0.1 (GOAL 6.20: SD 0.76 (CI 5.76–6.65) versus UC 6.1: SD 0.81 (CI 5.63–6.57)), less than the minimal clinically important difference (MCID) of 0.5. However, change from baseline was stronger in the intervention group: at 6 months the change in the emotions sub-score was 0.8 for intervention versus 0.2 for control. Costs were higher in the intervention group by £22.17.

Routine review with goal setting was considered more holistic, enhancing rapport and enabling patients to become active rather than passive participants in healthcare. However, time was a major barrier for nurses, who admitted to screening out patient goals they believed were unrelated to asthma.

**Conclusions:**

The difference in AQLQ score from baseline is larger in the intervention arm than the control, indicating the intervention may have impact if appropriately strengthened. The GOAL intervention changed the review dynamic and was well received by patients, but necessitated additional time, which was problematic in the confines of the traditional nurse appointment. Modification to recruitment methods and further development of the intervention are needed before proceeding to a definitive cluster randomised controlled trial.

**Trial registration:**

ISRCTN18912042. Registered on 26 June 2012.

**Electronic supplementary material:**

The online version of this article (doi:10.1186/s13063-016-1684-7) contains supplementary material, which is available to authorized users.

## Background

Asthma affects more than 300 million people globally [1, 2], with the United Kingdom (UK) accounting for an estimated 5 million of this total [3–6]. The financial cost to the health service is considerable [7–11], with the overall UK expenditure on asthma estimated at more than £1 billion per annum [5]. Self-management is important for improving health and well-being and reducing the economic burden of asthma [5, 7–28].

The current focus of asthma self-management is to provide sufferers with a written personalised asthma action plan (PAAP) [13, 14]. PAAPs focus on adherence to medication/monitoring and early recognition/remediation of exacerbations and are core components of effective self-management [29–33]. However, they do not incorporate the wider needs and views of the individuals who have to integrate the management of the disease into their daily lives. This exclusive medical focus can lead to a breakdown in communication and a failure of individuals with asthma to engage effectively in self-management [34, 35]. Divergent perceptions of asthma and how to manage it, and a mismatch between what patients want and need from plans and what is provided by professionals, are barriers to success [29, 30, 33]. To overcome these barriers, there is a need both to identify and address individual patient goals [36] in the wider context of their life and family [33]. Active patient engagement allied with appropriate self-management training of motivated and supported health professionals are key elements for any effective self-management intervention [28].

Goal setting is increasingly recognised as an effective behavioural technique for assisting patients with long-term conditions to improve health-related self-management behaviour [37–42]. As providers of self-management support, health professionals work with patients to identify goals that are important to the patient, that may be achievable and with which they can engage. They then help patients make specific action and coping plans to achieve those goals [43, 44]. The focus and content of the self-management discussion is central to the outcome [33, 45], as identification of these goals is often not easy.

Set within the Medical Research Council’s (MRC’s) framework for developing and evaluating complex interventions [46], we revised a previously developed draft intervention [45, 47] which involved proactive goal elicitation and prioritisation. Feedback from early use of this intervention led us to make two changes in finalising this novel intervention. Firstly, we enhanced the format within which it was presented in order to aid understanding and ease of use, and secondly we supplemented it with a stronger action/coping planning element delivered by a primary care-based asthma nurse during an asthma review consultation.

Our goal was to conduct a pilot cluster randomised controlled trial (cRCT) that used quantitative and qualitative methods to address two objectives: (1) to pilot the intervention and its delivery in routine primary care asthma clinics, and (2) to pilot the trial process including recruitment and performance of outcomes. More specifically, we sought to assess our ability (and refine approaches) to recruiting practices, practice staff and patients; establish the best way of delivering the intervention; assess the acceptability and perceived utility of the tool by both patients and health professionals; and finally gain sufficient information on outcomes to inform a sample size calculation for a subsequent trial.

## Methods

### Design, ethics and registration

We used mixed methods, combining a pragmatic parallel, single-blinded, multi-centre, cRCT design with an embedded qualitative appraisal. We compared Achieving Good Outcomes for Asthma Living (GOAL) with usual care (UC). Our 18-month feasibility pilot trial was conducted in primary care practices in two Scottish health regions. The study was approved by the East of Scotland Research Ethics Committee (REC Ref. No. 12/ES/0050, July 2012), and all participants (practice nurses and patients) provided their informed, written consent to participate. The study protocol has been previously published and provides detailed information on the study’s rationale and methods [48]. An additional file outlines the trial process (see Additional file 1).

### Participants and recruitment

Practice recruitment began in October 2012 and was completed by December 2012. It was facilitated by the research and clinical networks and personal contact with individual practices [49, 50]. Practices were eligible if they had an asthma clinic run by a nurse in possession of an accredited asthma diploma and who regularly reviewed and managed patients with asthma.

Patient recruitment began in January 2013 with an end of April completion target. Linked to the practice annual asthma review process, patients were eligible if they were adults (aged 18 years or older) with active asthma (defined as a coded diagnosis of asthma, plus a prescription for an asthma medication in the previous year) and were due an asthma review within the following 3 months. We excluded people with a new diagnosis of asthma (<1 year); patients with chronic obstructive pulmonary disease (COPD) or any other significant lung disease; those deemed to be unable to give consent because of a major medical, social or communication reason; and patients already taking part in an asthma-related clinical trial.

Eligible patients were sent their routine review reminder from their general practitioner, together with an additional letter telling them the practice was involved in the study and asking them to read the accompanying information leaflet and return the consent form if they were interested in participating. A researcher then contacted potentially interested patients to ensure they understood what the project entailed, re-affirm their consent and complete the baseline questionnaire. Their choice of telephone or postal completion of the follow-up questionnaires was recorded. Most participants opted to complete the follow-up questionnaires by telephone.

### Randomisation

Randomisation was at the practice level. Consenting practices were anonymised and allocated to GOAL or UC, stratifying by health region and practice size. Minimisation was conducted based on achieving optimum balance for practice list size (two strata: <6000 and ≥6000 patients currently registered). The reason for randomisation by area was to ensure an even distribution of intervention and control practices, as there is potential for geographical variation in asthma management strategies between health boards. For consenting practices, allocation was determined by the statistician within Tayside Clinical Trials Unit (TCTU), who implemented the minimisation process. Given the nature of the intervention and the role that health professionals played in delivering the intervention to patients, it was not possible to blind practices to their allocation arm.

### Process measures

In order to assess feasibility, we collected data on practice and nurse recruitment, along with recruitment and retention rates for participating patients, and data completion during the trial.

### Outcome measures

Outcomes were measured at 3 and 6 months after the date of the review at which the intervention was delivered, and were chosen to reflect both clinical and patient-centred priorities. Our primary outcome measure was the validated mini Asthma-related Quality-of-Life Questionnaire (mAQLQ) [51], which has 15 questions in four domains (i.e. symptoms, environment, emotions and activities). Secondary outcomes measured asthma control (Asthma Control Questionnaire (ACQ) [52] and health services resource use data); patient self-efficacy (Patient Enablement Instrument); and cost effectiveness (cost per quality-adjusted life years (QALY)), measured using the health-related quality of life measure EuroQol (EQ-5D-3 L) [53]. Data on identification of study participants, ease of recruitment and retention within the trial were also recorded. We had originally planned to conduct a value of information (VoI) analysis, but this did not prove possible due to recruitment problems.

### Study intervention

GOAL was underpinned by a combination of theoretical models related to self-management: goal setting theory [54], Leventhal’s Self-Regulation Model of Health and Illness [55] and the health action process approach [44]. GOAL was delivered in primary care by the practice asthma nurse. The intervention, reported using the Template for Intervention Description and Replication (TIDieR) format [56] and illustrated in the CONSORT flow diagram (see Additional file 1), consisted of:

1. Healthcare professional training: Nurses attended a half-day workshop on trial procedure covering an update on the components of a ‘standard’ asthma review (control assessment; peak expiratory flow (PEF); check of inhaler technique; review of medication, etc. [57]) delivered by a qualified asthma nurse (GH) and specific training in the use of the GOAL-elicitation tool and GOAL-setting process in the management of asthma delivered by the team of two nurse practitioners and a health psychology researcher (GH, AT and PA)..
2. GOAL and action planning: In addition to a standard asthma review, patients received the GOAL intervention as part of the review consultation. The GOAL-elicitation tool was posted to patients prior to their scheduled asthma consultation, and they were asked to complete it at home and bring it with them to the review. The tool asked patients to write down what they wanted to achieve in their daily life, the priority of each goal and the extent to which asthma made it difficult to achieve those goals. The information was then to be used during the review to underpin a focussed discussion about the meaning, importance and priority given to the goals, selecting a goal on which to focus and its barriers and facilitators. During the review meeting, an individualised action plan, based on selected goals, was then negotiated and agreed on with the practice nurse. This plan was offered in addition to any symptom-related PAAP normally provided by the practice.

As a pragmatic trial, we did not provide a detailed script; the nurses were allowed a degree of freedom to deliver the intervention and tailor it to patient/circumstantial needs [58].

### Usual care control group

The nurses attended the first session of the workshop relating to project procedure and asthma review only. Patients in the UC arm received a ‘standard’ asthma review.

### Quantitative process

#### Data collection

Data on quality of life, asthma control and patient self-efficacy (see Table 1) were collected from patients in both arms at baseline (after recruitment) and at 3 and 6 months post-intervention via telephone. The researchers contacted each participant at the pre-arranged time. If there was no reply, repeated attempts were made within the hours and days after the designated post-intervention period until each patient was contacted. Data on health services reseource use for the period 6 months pre- and post-intervention were collected from patient records, by researchers (PA and AT) visiting the general practitioner (GP) practices, after the final post-intervention questionnaires had been completed.

**Table 1:** Schedule of procedures during study period

	Baseline	Asthma review	6 weeks	3 months	6 months	Close of data collection
mAQLQ	x			x	x	
ACQ	x			x	x	
EQ-5D-3 L	x			x	x	
Patient enablement instrument	x			x	x	
GOAL tool		x				
Follow-up call			x			
Health services resource use data					x	
Qualitative interviews with health professionals						x
Qualitative interviews with patients						x

#### Sample size and statistical and economic analysis

No formal sample size calculation was undertaken for this feasibility pilot trial. Based on our previous experience working with asthma-trained nurses in primary care [59] and the published literature [60], we aimed to recruit 80 patients with active asthma from eight practices across two health boards (i.e. 10 patients per practice resulting in 40 in each arm) in the hope that this would allow sufficient data to estimate recruitment, compare the groups, calculate the intra-cluster correlation coefficient (ICC), estimate effect size, explore the acceptability of the goal-setting tool and be a small enough target for each practice to achieve with relative ease.

Although comparative analysis is not normally a factor in feasibility studies [61], we felt it was important to test the feasibility of our chosen primary outcome measure and estimate variance. The published protocol provides a detailed account of our analysis plan [48]. The primary analysis was based on adjusting all models for clustering within practices and stratifying by size and region. The differences in the validated mAQLQ scores between the intervention and control groups at baseline and 3 and 6 months post-intervention were compared. A clinically relevant improvement was an increase of ≥0.5 in individual mAQLQ mean scores [51]. Resources were valued, where possible, using unit costs from standard national sources. QALYs were estimated from the responses to the EQ-5D-3 L. Generalised linear models were used to estimate the mean difference in QALYs and costs adjusted for baseline differences. Robust cluster option was used to allow for clustering of observations by practice.

### Qualitative process

#### Data collection

Experienced qualitative researchers (PA and AT) conducted individual semi-structured interviews with practice nurses and patient participants. Our target was to interview at least one nurse from each of the 10 participating practices and a purposive sample (by age, gender, asthma severity and practice nurse) of 10 patient participants from both the GOAL and UC arms. All practice nurse interviews and the majority of patient interviews were conducted face to face (telephone interviews were offered as an option) with the interviews lasting 30–40 minutes. The interviews took place with patient participants after completion of their 6 month post-intervention telephone questionnaires, and for the nurses when they had undertaken all the trial reviews. The aim was to explore (1) experience, acceptability and perceived usefulness of the GOAL tool and goal-setting process; (2) perceived impact of the intervention on self-management, quality of life and clinical practice; (3) perceived change in professional-patient communication; and (4) experiences and acceptability of all elements of the trial.

#### Qualitative process evaluation

Interviews were audio recorded, transcribed verbatim and managed using QSR NVivo v10 [62], and analysed following the guidelines for thematic framework analysis [63] allowing for systematic classification and organisation of the data in terms of key themes and emergent patterns. The analytical process was an iterative one, with new issues identified during interim data analysis included in subsequent interviews. Additional information on the analysis process can be found in the published protocol [48]. The data were coded and checked for accuracy by the two researchers, who constantly searched for alternative explanations and discussed emerging themes and patterns with each other and the wider research and project management team. To aid interpretation of the data, we referred to the constructs of normalisation process theory (NPT) [64] and presented the results using the NPT headings.

## Results

### Process and feasibility

#### Quantitative data: recruitment and retention

**Fig. 1:**
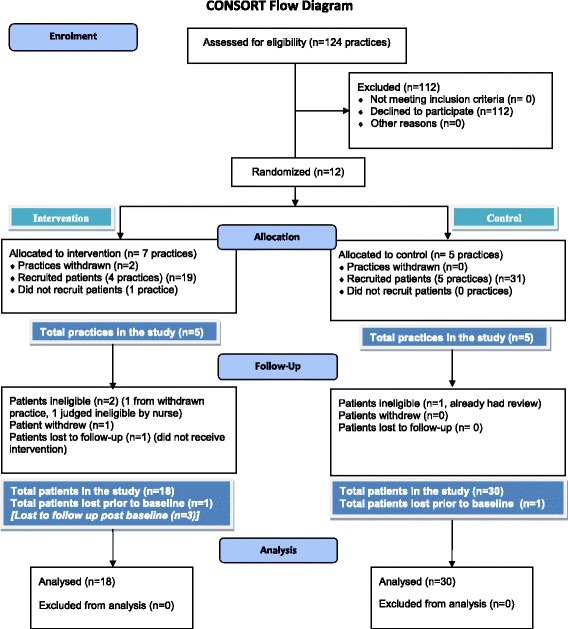
Consort flow diagram

The CONSORT diagram (Fig. 1) describes the participant flow through the study, the number of practices and patients recruited and intervention allocation and outcome. While the study exceeded the practice recruitment target of eight practices, we failed to achieve the patient recruitment target of 80 despite extending recruitment until the end of September 2013.

#### General practices and healthcare professionals

At the outset of the trial, 124 GP practices were approached to participate, and 19 (15%) declared an interest. Eleven were recruited and randomised (six intervention and five control). Two intervention practices withdrew after they had sent out the first tranche of study letters but before any patients had been reviewed by the nurse. Practices on the reserve list were approached, and one agreed to participate. Ten practices completed the study: five in the intervention arm and five in the control arm (see Table 2 and Fig. 1). Eleven nurses from nine practices attended the workshop. Those nurses who could not attend the workshop received one-on-one on-site training (*n* = 4).

**Table 2:** Practice recruitment by size and region

Region1	Small (<9000) and urban	Small (<9000) and rural	Large (≥9000) and urban	Large (≥9000) and rural	Total
	1	2	1	1	5
Region 2	Small (<6000) and urban	Small (<6000) and rural	Large (≥6000) and urban	Large (≥6000) and rural	Total
	0	3	2	0	5
Total	1	5	3	1	10

#### Adults with active asthma

The patient demographic data and characteristics are shown in Table 3. Fifty patients declared an interest in participating. Two were ineligible (one from a practice that had withdrawn from the trial and one who had been given their asthma review prior to baseline assessment), resulting in a total of 48 patients (30 UC and 18 GOAL). Retention of patients within the study was good; only three patients (6%, all from the intervention arm) were lost to follow-up. One, due to severe illness, could not engage in the intervention; another chose to withdraw from the study before their review consultation; the third, although keen to participate, was deemed by the nurse to be an unsuitable candidate for the intervention.

**Table 3:** Patient demographic data and baseline characteristics

Variable	Intervention		Control		Total		Qualitative sub-group
	*N*	%	*N*	%	*N*	%	*N* (%)
Area							
Region 1	10	(55.6%)	18	(60.0%)	28	(58.3%)	7 (50%)
Region 2	8	(44.4%)	12	(40.0%)	20	(41.7%)	7 (50%)
Total	18	(100.0%)	30	(100.0%)	48	(100.0%)	14 (100%)
Practice size							
Large	10	(55.6%)	12	(40.0%)	22	(45.8%)	7 (50%)
Small	8	(44.4%)	18	(60.0%)	26	(54.2%)	7 (50%)
Total	18	(100.0%)	30	(100.0%)	48	(100.0%)	14 (100%)
Rurality							
Urban	14	(77.8%)	7	(23.3%)	21	(43.8%)	8 (57%)
Rural	4	(22.2%)	23	(76.7%)	27	(56.3%)	6 (43%)
Total	18	(100.0%)	30	(100.0%)	48	(100.0%)	14 (100%)
Gender							
Male	9	(50.0%)	12	(40.0%)	21	(43.8%)	7 (50%)
Female	9	(50.0%)	18	(60.0%)	27	(56.3%)	7 (50%)
Total	18	(100.0%)	30	(100.0%)	48	(100.0%)	14 (100%)
Age (years)							
*N*	18		30		48		14
Missing	0		0		0		0
Mean	60.4		54.0		56.4		57.2
SD	18.01		13.39		15.42		17.6
95%-LCL	51.49		49.03		51.96		
95%-UCL	69.40		59.03		60.92		
Minimum	19		21		19		22
Q1	52.0		47.0		51.5		
Median	64.5		56.0		57.5		
Q3	75.0		62.0		67.0		
Maximum	84		76		84		81
BTS/SIGN treatment							
Step 1	1	(6%)	1	(3%)	2	(4%)	
Step 2	2	(11%)	14	(47%)	16	(33.5%)	2 (14%)
Step 3	13	(72%)	14	(47%)	27	(56.25%)	12 (86%)
Step 4	2	(11%)	1	(3%)	3	(6.25%)	

*SD* standard deviation, *LCL* lower confidence limit, *UCL* upper confidence limit, *BTS* British Thoracic Society, *SIGN* Scottish Intercollegiate Guidelines Network

### Qualitative data

Twenty-four interviews were conducted, with 10 practice nurses (one from each participating practice) and 14 patients (11 intervention and 3 control). Baseline characteristics of the qualitative sub-group are provided in Tables 4 and 5. Direct quotes illustrating the NPT domains are given in Table 6.

**Table 4:** Patient interview characteristics

Patient ID	Gender	Study group	Interview type	Age	BTS step	mAQLQ score		
						Baseline	3 mths	6 mths
101	F	I	Face-to-face	22	3	5.4	6.2	6.4
102	F	I	Face-to-face	62	3	6.8	6.6	6.8
103	M	I	Face-to-face	81	3	6.2	5.4	6.2
104	M	I	Face-to-face	52	3	6.6	6.2	6.4
107	F	I	Face-to-face	70	3	4.7	5.5	5.5
108	M	I	Telephone	79	3	5.0	5.2	6.7
901	F	I	Face-to-face	47	3	5.6	6.5	6.4
902	M	I	Face-to-face	67	3	4.5	6.0	5.1
1001	M	I	Telephone	75	3	5.6	–	–
1002	M	I	Face-to-face	57	3	4.3	4.2	4.4
1201	F	I	Face-to-face	55	3	5.2	4.8	5.6
1102	F	C	Face-to-face	62	2	6.0	6.8	6.5
805	F	C	Face-to-face	29	2	6.6	6.2	–
603	M	C	Face-to-face	43	3	6.5	6.6	–

**Table 5:** Details of health professional interviews

Nurse ID	Practice ID	Study group	Interviewed by
N101	01	I	PA
N102	01	I	PA
N103	01	I	PA
N09	09	I	PA
N10	10	I	PA
N08	08	C	AT
N06	06	C	AT
N11	11	C	AT
N03	03	C	AT
N12	12	I	AT

**Table 6:** Summary of findings as related to the NPT framework

NPT category	Patients	Professionals/practices
***Coherence: Meaning and sense making by participants***	After some initial uncertainty, many were able to set goals that ranged from everyday changes to more major challenges. The relevance of asthma to the goals was not always clear.	Nurses understood the concept of goal elicitation and understood its value but struggled with processing and prioritising the goal information and negotiating an action and coping plan within the timeframe of the review.
	**Patient 901 Female 47 years** *“….it was quite clear and the way it then broke down so you actually really had to think about what it is that's limiting ….. it did actually make you think much more deeply about how you went about these things and therefore what were the key things that were holding you back.”* **Patient: 103 Male 81 years** *“Yeah, it was easy to understand. The hard bit was to try and find an answer! ”* **Patient 1002 Male 57 years** *“Oh it was good. It was about what I do, about my lifestyle. So, yes, it was ….pertaining to my life and what asthma had done to it. ….it just came out, just flowed out.”* **Patient 901 Female 47 years** *“I thought it was very interesting, it was very difficult to work out what your goals were and then to think how asthma might affect them. Like, for instance, I've got ‘get fit’ there; I don't care about being fit, being fit is to enable me to do lots of other things, so I was getting confused with what was a goal, what did I really want to do and what were the mechanisms for getting there, which I think later on it pulled out, but on the first page I was getting very confused with those.”*	**Nurse 102** *“…it was good because it made them (patients) think more about how they felt.”* *“it was helpful in the sense that they (patients) told us a lot more than we would normally ask.”* **Nurse 103** *“it’s a good idea. It’s very thorough; it’s looking at the whole person and not just looking at the disease…”* *“…we tend to do the same core questions in the basic review…. I think it’s unlikely for us to be able to deal with other issues unless it’s been a very straightforward asthma review.”* *“I don’t think …we would really ever go into their life goals unless it cropped up, saying that ‘I really want to do this but I can’t because of my asthma’. … I don’t think is something that we’d… discuss, …probably due to time restraints…”*
***Cognitive participation: Commitment and engagement by participants***	Few patients volunteered for the study, perhaps reflecting unfamiliarity with the concept of goal setting, though 15 of the 18 patients participants remained committed throughout.	Nurses were enthusiastic about the concept which they perceived resonated with their role in providing self-management support.
	**Patient 101 Female 22 years** *“I have just finished university myself and had to do a research project and I'm more than happy to participate in anything that might make a difference to someone in the future.”* **Patient 103 Male 81years** *“I don’t mind taking part…I know when I had my pacemaker fitted I was asked a load of questions and I agreed…No, I think if I’m being helped then I return the help in some way … and I think that’s what one should do.”*	**Nurse 101** “we already do self-management plans and discuss their future plans and how to deal with their symptoms and how to deal with life in general …” **Nurse 3** *“I … think it's because I would like to…see their control better, try and make their quality of life better, and help the patient to understand what asthma is. It's their disease, it's their illness, and therefore make them able to manage it themselves.”* *“I've enjoyed it. … it was stressful at the very beginning…before we actually saw our first patients…but…I kind of settled down and thought, actually…there's nothing more to this than what I've been doing.”* **Nurse 6** *“…made me a bit more aware… looking more objectively at the patient and their work…it…changed my way of thinking…that was good.”*
***Collective action: The work participants do to make the intervention function***	Patients generally attempted to complete the pre-consultation goal setting exercise, but opinions about whether this was a useful task ranged from ‘insufficiently motivating’ ‘useful clarification’, ‘already clear about my goals’ ‘not sure I have/want goals’.	The organisational processes (exacerbated because of trial recruitment processes) were complex and did not go smoothly. The task of assimilating and discussing goals substantially increased the workload for the nurses who struggled with the demands on their time.
	**Patient 107 Female 70 years** *“…… it gave me an opportunity to sit down and think things out a bit more clearly …… it made me prioritise much more……. I had a lot more aims and goals before I narrowed it down……”*	**Nurse 10** *“….what was the process again? We got the amount of patients that were due and then there were certain criteria, that’s right. And then in the office they got the patients …. and then I had to go through the list and check it..…quite a lengthy process really trying to find the patients that were eligible…… we always seem to be behind in getting the letters out because the admin staff are that busy and I was that busy….”* **Nurse 102** *“It just took up a bit more time… I went over, because of the things that they put down, you know, I would never talk about losing weight, well I would talk about losing weight to asthma patients if they're very heavy and obese, but diet and stuff isn't something that I would bring up in an asthma clinic. So I went over because I talked about things.”*
***Reflexive monitoring: Participants reflect on or appraise the intervention***	The goal-focussed review was experienced as being more holistic, person-centred and partnership-based	Nurses did not believe that the goal-setting intervention significant changed how the review was conducted – though it substantially disrupted their appointment schedule.
	**Patient 1002 Male 57 years** *“…it brought to life what…nobody else has ever asked. it …opened my eyes to my asthma and now I think I’m in control of it rather than asthma being in control of me.”* **Patient 1201 Female 55 years** *“…basically what this has done is…reminded me that I don’t pay close enough attention to matching being good to myself, being kind to myself, with my conditions. And that I actually have to actively look after myself, not take it for granted, be sensible about using the medication appropriately, monitoring how I’m doing …[it] focused me on that.”* **Patient 101 Female 22 years** *“I think it changed what we’d done in our asthma appointments ….because it gave it more of a focus…which is quite important.”* **Patient 901 Female 47 years** *“…I thought it was very useful, it was like having a life coach on the NHS! It felt much more like a team, felt much more that she knows who I am and therefore we’re working together on my health rather than me doing what I'm told and being monitored.”*	**Nurse 10** *“… I think it’s quite lengthy…and …a bit difficult…it was all about his medication and his work….his goal was to be able to do his job without being so short of breath. I found it quite difficult actually…I don’t know if it was because [it’s]… down in black and white….what he wants…you kind of think oh God am I going to be able to actually sort for this for him…”*
***Implementation***		
	**Patient 901 Female 47 years** *“I suppose it’s difficult with studies but I suppose if it had been brought up rather than on quite a long letter, which it was wasn’t it, a reasonably long letter…and then there's forms to sign and there's a whole lot of barriers, and because it’s a study that's why it happens, but if it had been brought up perhaps when I’d gone in to see the nurse as a start off section, then that might have made people more responsive cause they didn't have to trawl their way through all the…words.”*	**Nurse 9** *“…I think it was the time when we first started … it was near the end of the financial year for the GPs then the start of the beginning, so a lot of our patients had been seen at the beginning of the year [spring/summer of the previous year] and it was only those that hadn’t been seen and they were down the line of possibly being exception coded anyway. So these were the patients that hadn’t turned up at the last two or three. So the selection wasn’t good.”* **Nurse 3** *“I did have an option but I didn’t really have an option because the GP who's interested in asthma had ticked the box and sent it off and then I probably got - I got told about two days before, by the way we've done this and we would like you to do it. So, in hindsight, I read the information…”* **Nurse 3** *“I think probably you know how we got the goal flier, I think putting that in and getting them just to contact myself to discuss it further… the patients were saying it was too wordy for them and they … I suppose it’s something unknown to them as well but they just didn't read it …too many words.”* **Nurse 8** *“…people weren’t too sure how connected it was with the practice…they knew they'd got an invitation from us but then they got this other thing from the University and they weren’t sure, they kind of came along to me to ask me was I involved in that, but by the time they'd asked me it was too late …if you could simplify the paperwork and the process of it for patients, or even the initial contact could even be ‘if you're interested in this contact the practice nurse’ and they would at least understand that it’s something we were involved in, because … they know that we manage their asthma and they're almost a bit suspicious of anybody else who might be coming in.”* **Nurse 3** *“…after the review I would ask them why they had decided not to take part. … I think they thought it was more than just a couple of phone calls…I think they thought there would be more into it. Whether they'd had - you know, they get so many questionnaires and things and projects to follow up and things like that, so I think they maybe thought it was more involved. …if …you had just a sheet of paper that said, this will involve a telephone call which will last ten minutes. You'll see your asthma nurse for a normal asthma [20:00] review and then we’ll follow it up afterwards…”*

#### Coherence: meaning and sense making by participants

Patient participants were positive about the GOAL process, with most reporting that the aim of the GOAL-eliciting tool was clear and that it was easy to understand and use. Some patients observed that the term ‘goal’ could, at first, be misleading, as it carried connotations of things that are unusual/exciting or those about to happen in the future. Nevertheless, despite initial difficulties, all patients reported eventually realising that ‘goal’ referred to anything they wanted to do regardless of its content and nature.

The extent to which patients thought the tool was relevant to them was influenced by beliefs about their life circumstances and their asthma. Patients with simple or clear goals, such as those relating to problematic or urgent physical symptoms (e.g. croaking voice, disturbed sleep, breathlessness at work) or to imminent life events (moving house, getting married), found their goals easy to articulate. Others initially struggled to articulate their goals; some believed there was little they wanted to achieve given their age and/or considered their asthma to be mild, had no clear idea about what they really wanted to achieve or struggled to understand the difference between a goal and the process towards achieving the goal.

The generated goals ranged in content (physical symptoms, functional problems, related to work, home and family, lifestyle, health and forthcoming life events) and nature (some referred to a desired future state, e.g. moving house or losing weight; while others referred to retention of a current state, e.g. continuing to work or remaining healthy). Unsurprisingly, the majority of goals referred to everyday or commonplace things rather than extraordinary achievements.

There was some feeling among patients that the goals they had thought of had little relevance to asthma either because patients believed their asthma to be mild and well controlled, because they thought that other health conditions such as arthritis had more of an impact on goal achievement or because they considered themselves ‘too old’ to have goals. Those with well-controlled asthma reported struggling to understand how setting and working towards a broader life goal was linked to asthma-specific goals. Some older patients and those who believed their asthma was not a problem perceived that this tool might be more relevant to younger people or to those with less well-controlled/or severe asthma.

From the health professional perspective, the process of goal elicitation was easy to understand. However, they found it difficult to produce an action and coping plan based on the information produced by the patient within the time frame of the consultation. They also struggled with the introduction of life goals into what was an asthma review. Changing the ‘normal’ review routine meant having to think laterally at times, and while they felt it was important to encourage patients to think about goals, there was a feeling that it complicated the asthma review process and led to a lengthier consultation.

#### Cognitive participation: commitment and engagement by participants

GOAL was delivered by the practice nurse and therefore depended on a high degree of commitment from the practice staff. Practice buy-in to the concept of using the GOAL tool for supporting the self-management process was evident — our target practice recruitment being achieved in both regions. The main driver of the intervention within the practice was the nurse, as it was he/she who would deliver the intervention. Their pre-study enthusiasm was apparent, as they perceived their role in asthma management to include self-management advice and action planning. Those patients who engaged with the project remained committed throughout, and the attrition rate was low — only three left before the end of the study as a result of ill health. The biggest problem was in ‘connecting’ with eligible patients to encourage them to engage with the project in the first place.

#### Collective action: the work participants do to make the intervention function

The success of the intervention was dependent on active engagement at a practice, nurse and patient level. Practices were asked to recruit patients by selectively sending the study information to eligible patients with the practice asthma review reminder letter. This did not always go smoothly.

Patients had to engage with the project, understand what was being asked of them and complete the questionnaire prior to attending their review. They reported mixed views about whether writing down their goals had been a useful exercise. Those who previously had a clear idea about their goals from the outset felt it had little impact on their thought processes. There was also some feeling that merely writing down the goals was not sufficient to motivate people to actually take steps to achieve them, unless it was subsequently translated into concrete actions. However, patients who had difficulty articulating their goals found the exercise quite useful in clarifying their goals and prioritising what they wanted out of life. The tool helped them distinguish between end-state and transitionary goals, streamlining them into a logical sequence.

Practice nurses had to understand the GOAL-elicitation process and construct a conversation within the review that would result in prioritisation of goals and creation of an action and coping plan. They found that being presented with the patient’s prioritised goals at the review did not always allow them time to assimilate the information prior to the discussion. The inclusion of what they perceived as ‘non-asthma’-related goals was felt to be a diversion from what they should be focussing on, and they also appeared to find it difficult to construct a negotiated action and coping plan within the time frame of the review. The work associated with the intervention was therefore regarded by most nurses as considerable, particularly as it was performed in addition to ‘normal processes’.

No specific actions were expected from either the health professionals or the patients post-study. The lack of follow-up limited the information on longer term impact of the intervention, both for patient self-management and for the structure and focus of the review conversation.

### Reflexive monitoring: participants reflect on or appraise the intervention

Patients believed that the intervention made them pay greater attention to their asthma/health, affirming the importance of good self-management and motivating them to take an active part in managing their health. It focussed their attention on small things that affect/are affected by asthma, which often remain at a subconscious level and were therefore ignored. For those who could not think of any goals, it affirmed their well-being and absence of problems/issues, which was considered valuable in its own right.

For some patients, the intervention changed the nature of their asthma review. It seemed to provide them with an opportunity to raise a number of issues they had never been asked about before and would not have otherwise reported. Unlike the usual asthma reviews, often described as ‘output checks’ and ‘perfunctory’, the goal-focussed review was experienced as being more holistic, person-centred and partnership-based. It made them feel special, improved their rapport with the nurse, enabled them to discuss issues unrelated to asthma and helped them feel active partners in their care. From the health professional perspective, it helped the nurses know the patients on a personal level, consider the wider context of their life, address issues not directly related to asthma and be more likely to follow those issues up in subsequent visits.

However, although patients experienced a positive change in review content, nurses did not believe that the goal-setting intervention brought a significant change to how the review was conducted. There was an acknowledgement by some nurses that having the goals documented formally meant that they were definitely attended to and discussed, but there was a general feeling (in contrast to the views of patients) that the intervention did not make them do anything differently. The most significant impact the intervention was seen to have on their clinical practice was lengthening consultations with a negative effect on their appointment schedule.

#### The potential for implementation

Nurses considered the GOAL tool easy to understand and use; however, the GOAL action and coping plan was poorly implemented. While patients thought that it improved the quality and focus of the review, health professionals felt that it was an unnecessary additional burden during the review, particularly as they felt they were already ‘doing this’.

The GOAL-eliciting tool provided patients with an opportunity to raise a number of issues during the asthma review that would not previously have been reported or otherwise explored. There was a feeling that the tool gave them a voice to make their issues and problems known, providing a structure for a focussed conversation — something which they felt was lacking in the normal routine review.

Nurses recognised that the GOAL tool highlighted a broad spectrum of issues (lifestyle, work, social, etc.) never normally discussed in the context of a routine review. This provided an insight into patients’ reasoning behind their goals, helping them identify issues which may need addressing before focussing on a goal to prioritise in the action plan. However, there was some contention that, rather than eliciting new information, the GOAL tool merely helped structure the conversation and provided a formal status to the goal information, making it more likely that it would be addressed. They conceded that it had changed what they asked during the review and made them more aware of the need to include the wider aspect of patients’ lives.

Despite the fact that patients liked the time and attention GOAL afforded them, the practicalities to organisation and time were seemingly major barriers to the use of the tool in practice.

### Outcomes

Because of an extension to the recruitment period, we were only able to collect 6 months post-intervention data on 28 of the 48 patients. For an additional 13 patients, we had 3 months post-intervention data, and for 7 patients, only baseline data were available. Collection of health service resource data was restricted to patients who had 6 months post-intervention information available (28 patients: 14 GOAL and 14 UC).

At 6 months post-intervention, disease-specific quality of life (primary outcome) as measured by the mAQLQ, was marginally higher by a mean of 0.1 in the intervention group compared to the control (GOAL 6.20: SD 0.76 (CI 5.76–6.65) versus UC 6.10: SD 0.81(CI 5.63–6.57)). This was less than the minimal clinically important difference (MCID) of 0.5. However, the change from baseline to 6 months was stronger in the intervention arm: the difference in total and symptom scores in the intervention group from baseline was 0.4 compared with 0.2 in the control arm. The largest change was in the emotion sub-score with a difference from baseline to 6 months of 0.8 for the intervention and 0.2 for the control. Environment and activity also improved, to a lesser degree. An additional file shows this in more detail (see Additional file 2). QALYs were lower by 0.027 and costs higher by £22.17 in the intervention group compared to the control. An additional file provides more detail on the cost analysis (see Additional file 3).

## Discussion

### Main findings

Our study evaluated the acceptability, effectiveness and cost-effectiveness of a GOAL-elicitation tool for people with asthma managed in primary care and the feasibility of conducting a mixed methods cRCT. Trial data collection was feasible, and we achieved data saturation with respect to our key qualitative objectives of understanding the potential for implementation. However, despite successfully recruiting the required number of practices, we did not achieve our target for patient recruitment. Overall, the intervention resulted in only modest increases in asthma-specific quality of life which were well short of the MCID. Recruitment difficulties and organisational barriers for implementation mean that a full trial of the tool in its present format would be premature and likely to fail.

Patients and nurses were generally positive about the benefits of goal elicitation, though nurses struggled to merge the goal activity with their normal review process. Some patients struggled to identify issues they wanted to prioritise, and nurses felt they had little time to prepare to discuss the patients’ agendas. Contrary to patient perceptions that the GOAL-eliciting tool focussed attention on issues that would otherwise not be addressed, nurses, although acknowledging that the tool gave structure to the conversation around goals, did not believe the intervention significantly changed how the review was conducted.

### Strengths and limitations of the study

Use of the NPT model [64] to analyse the data helped us understand how the intervention might be accepted, the facilitators and barriers to implementation and whether any difficulties could be overcome. Active patient engagement and training of motivated and supported health professionals are essential elements for any effective self-management intervention [28, 43, 44]. A major strength of our study was that it tested a theoretically derived intervention that had already shown promise in scoping studies [45, 47]. The study built on extensive development and qualitative work with nurses and patients and adopted a systematic approach to designing and piloting a complex intervention [45–47].

The robustness of the practice recruitment process (facilitated as it was by local research and clinical networks and by personal contact with individual practices) and the randomisation process (carried out by a clinical trials unit) ensured that we achieved our target of 10 practices, five in each arm of the study. However, it became clear that the time allocated for practice recruitment was insufficient, coming as it did during a very busy time of the clinical calendar. This affected not only our ability to arrange meetings to discuss the project with members of the practice team, but also the timing of the project training, which had to be delayed until the New Year.

In contrast, despite adhering to the published evidence for maximising success when conducting and recruiting for a trial of a self-management intervention in primary care [28, 50, 65], patient recruitment was well below our set target of 80, which has consequences for the future conduct of a full trial. An additional conundrum was the differential of patient engagement/recruitment between the two arms (18 versus 30) despite the even spread of practices. Our lack of success in recruiting patients meant we failed to generate enough quantitative data to conduct robust statistical analysis. This was compounded by the fact that the delayed start and an extension to the recruitment period meant that many recruited patients were not in the study long enough to complete the 6 months follow-up questionnaires. Attrition, however, was low perhaps due to (1) the fact that patients who agree to participate in such studies are highly motivated, and (2) the benefits the recruited patients felt they received from the process.

The difficulties we faced in patient recruitment for the trial mirror the challenges faced by other pragmatic primary care studies [50, 66, 67]. However, although a major issue, this did not affect our ability to identify and interview an adequate number and range of patient participants in the qualitative study. The range of perspectives within the multi-disciplinary research team ensured the data were interpreted in a balanced and methodical way underpinned by theoretical knowledge.

### Interpretation of findings

#### The challenge of undertaking trials of complex interventions

Recruitment to trials within primary care settings is known to be challenging, with one survey finding that approximately one-third recruited to timetable, one-third required up to 50% more time than planned and another third required more than 50% extra time than originally planned [67]. Despite the use of robust and previously tested recruitment strategies in our study [48–50], patient recruitment was problematic, with only one practice achieving their target of 10 patients. This practice did not report any difference in their approach to patient recruitment or the way they conducted the intervention; success was attributed by their lead GP to the fact that they were a large, urban and research active practice. The patients from this practice were used to requests for participation in research, and the staff maintained a ‘whole practice’ commitment to research projects they agreed to take part in. This last point, an important documented facilitator for the effective execution of a trial [68–70], may have been the catalyst to their success.

Our practices were recruited from research networks, clinical networks and via personal contact [49], and any lack of communication and agreement within the practice team is believed to have an impact on enthusiasm for the study process [50, 68]. In addition, from a patient perspective, any perceived lack of connection between the study and their medical management due to unfamiliarity with the concept of research and the research team reduces the likelihood of recruiting them to the trial [66, 69]. The success of any future trial may necessitate limiting practice recruitment to those with extensive experience of research participation [71]. Alternatively, to encourage whole practice engagement, the structure and substance of the pre-intervention training programme would need to be more robust and involve all members of the practice team [71] — a challenging prospect in UK primary care today.

We took cognisance of published advice on effective ways to recruit and retain participants including meeting face to face with practice managers, GPs and practice nurses, developing a rapport and maintaining engagement throughout the study [49, 50, 66, 71]. A project logo was designed to brand the study and help it stand out. Patient participants were given options for questionnaire completion; a telephone helpline was established, and we were very flexible with dates and times for contact to complete the questionnaires. However, ethical requirement for an overly wordy study information sheet and prohibition of follow-up telephone calls were key obstacles to patient recruitment [66, 69, 71].

#### The challenge of implementing complex interventions

Practice nurse pre-conceived beliefs about their ability to incorporate patient goals into the normal review process were a potential barrier to acceptance of the intervention, particularly as they had an impact on the length of the consultation. With no single route to optimum shared decision making, remaining flexible in approach and being open to new ideas is critical [72].

The GOAL-elicitation tool was generally welcomed by patients as an effective health behaviour change technique [38–40]. By making the asthma review more holistic, structured and focussed, the tool improved their ability to communicate and increased their belief in becoming more active self-managers [43, 44]. However, the need for self-management strategies was related to perceived severity of disease [29, 30, 33, 34]; the general view was that the intervention should be targeted to people with more severe asthma.

From the practice nurse perspective, the tool was seen to formalise patients’ goals rather than helping to elicit new information. It was not thought to bring a significant change into their clinical practice and review process other than lengthening consultation time [73]. Viewing the patient’s completed GOAL questionnaire for the first time at the review meant the nurses had very little time to assimilate the information before discussing goal prioritisation with the patient, and in many cases this resulted in poor completion of a negotiated and tailored action and coping plan. Our findings reinforce the need to give the nurses time during pre-intervention training to critically reflect on their current provision of patient-centred self-management support [33, 45] and the constructs of true shared decision making based on a patient’s own goals [43, 44]. A change in the way patients present their goals prior to the review would also have to be considered [72].

### Implication for future research and practice

Our results indicate that there would be major challenges in delivering a full trial of the GOAL intervention without substantial further refinement of the study recruitment and intervention process [48].

#### How does the intervention need to change?

Using a formalised process to identify issues and possible solutions [74], it was clear that the logistics of the intervention need to be less intrusive on the regular review process. To do this we need to look at alternative ways of implementing GOAL within the current restraints of general practice with more attention paid to a whole practice team approach with administrative staff included in the training plan. In addition, we need to simplify the GOAL-elicitation process to increase understanding and ensure clarity about what exactly is required.

#### How do the trial procedures need to change?

There needs to be a discussion with the medical ethics committee to allow for a more flexible approach to the patient information and recruitment pathway. In addition, the inclusion criteria need to focus on people with more ‘problematic’ asthma.

We need to:

- Allow the nurses the opportunity to review patient goals before the review
- Reduce the impact of the intervention on appointment time
- Improve the information flow and communication/agreement within the practice team
- Find ways to improve health professionals’ and patients’ familiarity with the concept of research and the research team or alternatively target only those practices that are part of a research network and regularly take part in research studies.

## Conclusions

This work is of particular relevance to the engagement of patients in self-management of a particularly important and prevalent condition (asthma). The findings confirm that recruiting for a trial in primary care is complex and difficult [28, 50, 66, 71]. Practices appear interested, but incorporation of a time-consuming intervention into their already busy workload is a major barrier to implementation [68]. However, with self-management now a policy as well as a clinical imperative [14], the need to provide evidence for good clinical practice means that it is essential that we find a robust way to test novel interventions of this type in primary care [28, 33, 46].

The study was too small to make a statistical case; however, the larger change from baseline in the intervention group compared with the control indicates the possibility of some potential benefit to patient quality of life, particularly in relation to the participants’ emotional state. However, major refinement of the study process is necessary before committing to a definitive cRCT. The design of the full trial must focus on reducing the perceived burden of the intervention on healthcare staff and be targeted towards people with moderate to severe asthma. The workload within primary care is unlikely to improve; therefore, a way has to be found to implement goal setting within current constraints. Improved branding, a ‘whole practice team’ approach and improvement in the quality and length of the training for those delivering the intervention would increase understanding of the value and potential of GOAL, helping to break down perceived barriers. A phased rollout with the addition of constructive feedback would allow them to try the intervention with a few patients to get used to it. In addition, training of support staff via an on-line platform may enhance and improve the sense of ownership of the process within the team. Alternative strategies for delivering the intervention such as the ‘brief advice’ approach taken by smoking cessation, complemented by a lay-led support service for those who need/want it, will need to be considered. In addition, streamlining of the GOAL-elicitation process using Internet technology solutions would emulate the progress being made for delivery of patient‐reported outcome measures (PROMs) in other long-term conditions such as cancer. Any future trial will be subject to further feasibility testing on the redesigned process.

## Additional files

Additional file 1: Shows the flow of participants through the trial according to the requirements of the CONSORT checklist. (DOCX 319 kb)
Additional file 2: Summarizes the change in mAQLQ (primary outcome) at 3 months and 6 months post-intervention in the intervention and control groups. (DOCX 47 kb)
Additional file 3: Provides a breakdown of health service resource use costs in the intervention and control groups. (DOCX 53 kb)

## Abbreviations

ACQ: Asthma Control Questionnaire
CI: Confidence interval
COPD: Chronic obstructive pulmonary disease
cRCT: Cluster randomised controlled trial
ICC: Intra-cluster correlation coefficient
mAQLQ: Mini Asthma-related Quality-of-Life Questionnaire
MCID: Minimal clinically important difference
MRC: Medical Research Council
NPT: Normalisation process theory
PAAP: Personalised asthma action plan
PEF: Peak expiratory flow
QALY: Quality-adjusted life years
REC: Research Ethics Committee
SD: Standard deviation
TCTU: Tayside Clinical Trials Unit
TIDieR: Template for Intervention Description and Replication
UC: Usual care
UK: United Kingdom

## References

[1] Masoli M, Fabian D, Holt S, Beasley R (2004). The global burden of asthma: executive summary of the GINA Dissemination Committee report. Allergy.

[2] Anandan C, Nurmatov U, van Schayck OCP, Sheikh A (2010). Is the prevalence of asthma declining? Systematic review of epidemiological studies. Allergy.

[3] British Thoracic Society (2006). The burden of lung disease.

[4] Health and Social Care Information Centre. Quality and Outcomes Framework: GP practice results. 2014. http://www.nhsemployers.org/your-workforce/primary-care-contacts/general-medical-services/quality-and-outcomes-framework. Accessed 30 June 2014.

[5] Asthma UK. Asthma facts & FAQs. 2014. https://www.asthma.org.uk/about/media/facts-and-statistics/. Accessed 30 June 2014.

[6] Mukherjee MGR, Farr A, Heaven M, Stoddart A, Nwaru BI, Fitzsimmons D, Chamberlain G, Bandyopadhyay A, Fischbacher C, Dibben C, Shields M, Phillips C, Strachan D, Davies G, McKinstry B, Sheikh A (2014). Estimating the incidence, prevalence and true cost of asthma in the UK: secondary analysis of national stand-alone and linked databases in England, Northern Ireland, Scotland and Wales-a study protocol. BMJ Open.

[7] Weiss KB, Buist AS, Sullivan SD (2000). Asthma’s impact on society: the social and economic burden.

[8] Yelin E, Katz P, Balmes J, Trupin L, Earnest G, Eisner M, Blanc P (2006). Work life of persons with asthma, rhinitis, and COPD: a study using a national, population-based sample. J Occup Med Toxicol.

[9] Gupta R, Sheikh A, Strachan DP, Anderson HR (2004). Burden of allergic disease in the UK: secondary analyses of national databases. Clin Exp Allergy.

[10] Anandan C, Gupta R, Simpson CR, Fischbacher C, Sheikh A (2009). Epidemiology and disease burden from allergic disease in Scotland: analyses of national databases. J R Soc Med.

[11] Simpson CR, Sheikh A (2010). Trends in the epidemiology of asthma in England: a national study of 333,294 patients. J R Soc Med.

[12] Accordini S, Bugiani M, Arossa W, Gerzeli S, Marinoni A, Oliveri M (2006). Poor control increases the economic cost of asthma: a multicentre population-based study. Int Arch Allergy Immunol.

[13] Global Initiative for Asthma (GINA). Global strategy for asthma management and prevention. Global Initiative for Asthma. 2012. http://ginasthma.org/. Accessed 10 Nov 2015.

[14] British Thoracic Society & the Scottish Intercollegiate Guidelines Network. British Guideline on the Management of Asthma. Guideline No. 141. 2014. http://www.sign.ac.uk/guidelines/published/numlist.html. Accessed 10 Nov 2015.

[15] Taylor S, Pinnock H, Epiphaniou E, Pearce G, Parke H, et al. A rapid synthesis of the evidence on interventions supporting self-management for people with long-term conditions: PRISMS - Practical Systematic Review of Self-Management Support for long-term conditions. Health Serv Deliv Res. 2014;2(53). doi:10.3310/hsdr02530. 25642548

[16] Gibson PG, Powell H, Coughlan J, Wilson AJ, Abramson M, Haywood P, Bauman A, Hensley MJ, Walters EH (2003). Self-management education and regular practitioner review for adults with asthma. Cochrane Database Syst Rev.

[17] Tapp S, Lasserson TJ, Rowe BH. Education interventions for adults who attend the emergency room for acute asthma. Cochrane Database of Systematic Reviews 2007, Issue 3. Art. No.: CD003000. doi:10.1002/14651858.CD003000.pub2. 10.1002/14651858.CD003000.pub2 PMC11491197 17636712

[18] Powell H, Gibson GP. Options for self-management education for adults with asthma. Cochrane Database Syst Rev. 2003;(1):CD004107. 10.1002/14651858.CD004107 PMC8406716 12535511

[19] Toelle B, Ram FSF. Written individualised management plans for asthma in children and adults. Cochrane Database Syst Rev. 2004;(2):CD002171. 10.1002/14651858.CD002171.pub2 15106169

[20] Lefevre F, Piper M, Weiss K, Mark D, Clark N, Aronson N (2002). Do written action plans improve patient outcomes in asthma? An evidence-based analysis. J Fam Pract.

[21] Boyd M, Lasserson TJ, McKean MC, Gibson PG, Ducharme FM, Haby M. Interventions for educating children who are at risk of asthma-related emergency department attendance. Cochrane Database Syst Rev. 2009(2). CD001290. doi: 10.1002/14651858.CD001290.pub2. 10.1002/14651858.CD001290.pub2 PMC7079713 19370563

[22] Bravata D, Gienger AL, Holty JE, Sundaram V, Khazeni N, Wise PH (2009). Quality improvement strategies for children with asthma: a systematic review. Arch Pediatr Adolesc Med.

[23] Lahdensuo A (1999). Guided self management of asthma—how to do it. BMJ.

[24] Kennedy A, Reeves D, Bower P, Lee V, Middleton E, Richardson G, Gardner C, Gately C, Rogers A (2007). The effectiveness and cost effectiveness of a national lay-led self care support programme for patients with long-term conditions: a pragmatic randomised controlled trial. J Epidemiol Community Health.

[25] Gordon C, Galloway T (2008). Review of findings on Chronic Disease Self-Management Program (CDSMP) outcomes: physical, emotional & health-related quality of life, healthcare utilization and costs, Centers for Disease Control and Prevention and National Council on Aging.

[26] Ring N, Jepson R, Hoskins G, Wilson C, Pinnock H, Sheikh A, Wyke S. Understanding what helps or hinders asthma action plan use: a systematic review and synthesis of the qualitative literature. Patient Educ Couns. [online publication] 2011;85(2). doi: 10.1016/j.pec.2011.01.025. 10.1016/j.pec.2011.01.025 21396793

[27] Sawyer S (2002). Action plans, self monitoring and adherence: changing behaviour to promote better self management. Med J Aust.

[28] Pinnock H, Epiphaniou E, Pearce G, Parke HL, Greenhalgh T, Sheikh A, Griffiths CJ, Taylor SJC. Implementing supported self-management for asthma: a systematic review of implementation studies. BMC Medicine. 2015;13(127). doi:10.1186/s12916-015-0361-0. 10.1186/s12916-015-0361-0 PMC4465463 26032941

[29] Brown R (2001). Behavioural issues in asthma management. Pediatr Pulmonol.

[30] Jones A, Pill R, Adams S (2000). Qualitative study of views of health professionals and patients on guided self-management plans for asthma. BMJ.

[31] Hoskins G, McCowan C, Donnan P, Friend J, Osman L (2005). Results of a national asthma campaign survey of primary care in Scotland. Int J Qual Health Care.

[32] Pinnock H, Thomas M, Tsiligianna I (2010). The International Primary Care Respiratory Group (IPCRG) Research Needs Statement 2010. Prim Care Resp J.

[33] Ring N, Jepson R, Pinnock H, Wilson C, Hoskins G, Sheikh A, Wyke S. Developing novel evidence-based interventions to promote asthma action plan use: a cross-study synthesis of evidence from randomised controlled trials and qualitative studies. Trials. https://trialsjournal.biomedcentral.com/articles/10.1186/1745-6215-13-216. 10.1186/1745-6215-13-216 PMC3561124 23164151

[34] Andrews K, Jones SC, Mullan J (2014). Asthma self management in adults: a review of current literature. Collegian.

[35] Royal College of Physicians (2014). Why asthma still kills: the National Review of Asthma Deaths (NRAD) Confidential Enquiry report.

[36] Deci E, Ryan RM (2000). The ‘what’ and ‘why’ of goal pursuits: human needs and the self-determination of behavior. Psychol Inquiry.

[37] Clark NM, Gong M (2000). Management of chronic disease by practitioners and patients: are we teaching the wrong things?. BMJ.

[38] Bandura A (1977). Self-efficacy: toward a unifying theory of behavioural change. Psychol Rev.

[39] Hardeman W, Johnston M, Johnston DW, Bonetti D, Wareham NJ, Kinmonth AL (2002). Application of the theory of planned behaviour change interventions: a systematic review. Psychol Health.

[40] Floyd D, Prentice-Dunn S (2000). A meta-analysis of research on protection motivation theory. J Appl Sol Psych.

[41] Scobbie L, Dixon D, Wyke S (2011). Goal setting and action planning in the rehabilitation setting: development of a theoretically informed practice framework. Clin Rehabil.

[42] Boa S, Wyke S, Haraldsdottir E, Duncan E (2014). Development, implementation and evaluation of a theory based goal setting framework for use in palliative care (G-AP-PC). Support Palliat Care.

[43] Gollwitzer PM, Sheeran P, Zanna MP (2006). Implementation intentions and goal achievement: a meta-analysis of effects and processes. Advances in experimental social psychology.

[44] Schwarzer R (2008). Modeling health behavior change: how to predict and modify the adoption and maintenance of health behaviors. Appl Psychol Int Rev.

[45] Williams B, Steven K, Sullivan F (2011). Tacit and transitionary: an exploration of patients’ and primary care health professionals’ goals in relation to asthma. Soc Sci Med.

[46] Medical Research Council. Developing and evaluating complex interventions. 2010. https://www.mrc.ac.uk/documents/pdf/complex-interventions-guidance/. Accessed 10 Nov 2015.

[47] Steven K, Sullivan FM, Williams B, Hoskins G. Integrating three perspectives of goals in asthma management: the patient the health professional and the evidence to improve asthma morbidity. CSO Focus on Research, Ref:CZF/1/8 2007.

[48] Hoskins G, Abhyankar P, Taylor AD, Duncan E, Sheikh A, Pinnock H, van der Pol M, Donnan PT, Williams B (2013). Goal-setting intervention in patients with active asthma: protocol for a pilot cluster-randomised controlled trial. Trials.

[49] Campbell M, Snowden C, Francis D, Elbourne D, McDonald AM, Knight R, Entwistle V, Garcia J, Roberts I, Grant A (2007). (the STEPS group). Recruitment to randomised trials: strategies for trial enrolment and participation study. The STEPS study. Health Technol Assess.

[50] Treweek S, Mitchell E, Pitkethly M, Cook J, Kjeldstrøm M, Johansen M, Taskila TK, Sullivan F, Wilson S, Jackson C, Jones R, Lockhart P. Strategies to improve recruitment to randomised controlled trials (Review). The Cochrane Collaboration. 2010(4). doi: 10.1002/14651858.MR000013.pub4. 10.1002/14651858.MR000013.pub5 20393971

[51] Juniper EF, Guyatt GH, Cox FM, Ferrie PJ, King DR (1999). Development and validation of the Mini Asthma Quality of Life Questionnaire. Eur Respir J.

[52] Juniper EF, Svensson K, Mork AC, Stahl E (2005). Measurement properties and interpretation of three shortened versions of the Asthma Control Questionnaire. Respir Med.

[53] Brooks R (1996). EuroQol: the current state of play. Health Policy.

[54] Locke E, Latham GP (1990). A theory of goal setting and task performance.

[55] Leventhal H, Brissette I, Leventhal EA, Cameron LD, Leventhal H (2003). The common-sense model of self-regulation of health and illness. The self-regulation of health and illness behaviour.

[56] Hoffmann T, Glasziou PP, Boutron I, Milne R, Perera R, Moher D, Altman DG, Barbour V, MacDonald H, Johnston M, Lamb SE, Dixon-Woods M, McCulloch P, Wyatt JC, Chan AW, Michie S (2014). Better reporting of interventions: template for intervention description and replication (TIDieR) checklist and guide. BMJ.

[57] Pinnock H, Fletcher M, Holmes S, Keeley D, Leyshon J, Price D, Russell R, Versnel J, Wagstaff B (2010). Setting the standard for routine asthma consultations: a discussion of the aims, process and outcomes of reviewing people with asthma in primary care. Prim Care Respir J.

[58] Thorpe KE, Zwarenstein M, Oxman AD, Treweek S, Furberg CD, Altman DG, Tunis S, Bergel E, Harvey I, Magid DJ, Chalkidou K (2009). A pragmatic–explanatory continuum indicator summary (PRECIS): a tool to help trial designers. J Clin Epidemiol.

[59] Hoskins G, Williams B, Jackson C, Norman P, Donnan P (2011). Assessing asthma control in UK primary care: use of routinely collected prospective observational consultation data to determine appropriateness of a variety of control assessment models. BMC Fam Pract.

[60] Billingham SA, Whitehead AL, Julious SA (2013). An audit of sample sizes for pilot and feasibility trials being undertaken in the United Kingdom registered in the United Kingdom clinical research network database. BMC Med Res Methodol.

[61] Lancaster GA, Dodd S, Williamson PR (2004). Design and analysis of pilot studies: recommendations for good practice. J Eval Clin Pract.

[62] Richards L (1999). Using NVivo in qualitative research.

[63] Ritchie J, Spencer L, Bryman A, Burgess RG (1994). Qualitative data analysis for applied policy research. Analysing qualitative data.

[64] May C (2006). A rational model for assessing and evaluating complex interventions in health care. BMC Health Serv Res.

[65] Campbell M, Elbourne DR, Altman DG (2004). for the CONSORT Group. CONSORT statement: extension to cluster randomised trials. BMJ.

[66] Malhotra S, Musgrave S, Pinnock H, Price D, Ryan D (2012). The challenge of recruiting in primary care for trial of telemonitoring in asthma: an observational study. Pragmatic Observational Res.

[67] Bower P, Wilson S, Mathers N (2007). How often do UK primary care trials face recruitment delays?. Fam Pract.

[68] Johnston S, Liddy C, Hogg W, Donskov M, Russell G, Gyorfi-Dyke E (2010). Barriers and facilitators to recruitment of physicians and practices for primary care health services research at one centre. BMC Med Res Methodol.

[69] Bell-Syer S, Moffett J (2000). Recruiting patients to randomized trials in primary care: principles and case study. Fam Pract.

[70] Bower P, Wallace P, Ward E (2009). Improving recruitment to health research in primary care. Fam Pract.

[71] Adams M, Caffrey L, McKevitt C (2015). Barriers and opportunities for enhancing patient recruitment and retention in clinical research: findings from an interview study in an NHS academic health science centre. Health Res Syst.

[72] Moser A, Houtepen R, Widdershoven G (2007). Patient autonomy in nurse-led shared care: a review of theoretical and empirical literature. J Adv Nurs.

[73] Bodenheimer T, Handley MA (2009). Goal-setting for behavior change in primary care: an exploration and status report. Patient Educ Couns.

[74] Bugge C, Williams B, Hagen S, Logan J, Glazener C, Pringle S, Sinclair L (2013). A process for Decision-making after Pilot and feasibility Trials (ADePT): development following a feasibility study of a complex intervention for pelvic organ prolapse. Trials.

