# Supplementary material for: Achieving Good Outcomes for Asthma Living (GOAL): mixed methods feasibility and pilot cluster randomised controlled trial of a practical intervention for eliciting, setting and achieving goals for adults with asthma
Source: Trials. 2016 Dec 8;17:584. doi: 10.1186/s13063-016-1684-7 (PMC5146838; doi:10.1186/s13063-016-1684-7)
Supplement: Additional file 1: — Shows the flow of participants through the trial according to the requirements of the CONSORT checklist. (DOCX 319 kb) [file 13063_2016_1684_MOESM1_ESM.docx]

Gaylor Hoskins, Purva Abhyankar, Anne D Taylor, Edward Duncan, Aziz Sheikh, Hilary Pinnock, Marjon van der Pol, Peter T Donnan and Brian Williams.

**“Goal-setting intervention in patients with active asthma: protocol for a pilot cluster-randomised controlled trial.”**

Trials 2013; 14:289. <http://www.trialsjournal.com/content/14/1/289>
