# Supplementary material for: Achieving Good Outcomes for Asthma Living (GOAL): mixed methods feasibility and pilot cluster randomised controlled trial of a practical intervention for eliciting, setting and achieving goals for adults with asthma
Source: Trials. 2016 Dec 8;17:584. doi: 10.1186/s13063-016-1684-7 (PMC5146838; doi:10.1186/s13063-016-1684-7)
Supplement: Additional file 2: — Summarizes the change in mAQLQ (primary outcome) at 3 months and 6 months post-intervention in the intervention and control groups. (DOCX 47 kb) [file 13063_2016_1684_MOESM2_ESM.docx]

**Additional File 2**

Summary of change in mAQLQ (primary outcome) at 3 months and 6 months in Intervention Practices and Controls

|  |  | **Intervention** | | | | | | **Controls** | | | | | |
| --- | --- | --- | --- | --- | --- | --- | --- | --- | --- | --- | --- | --- | --- |
| **Outcome** | **Time Point** | **N** | **Mean** | **SD** | **Median** | **lower CL** | **upper CL** | **N** | **Mean** | **SD** | **Median** | **lower CL** | **upper CL** |
| Change in Total Mini-AQLQ Score | 3 months | 14 | 5.82 | 0.831 | 6.03 | 5.344 | 6.304 | 27 | 5.80 | 1.204 | 6.13 | 5.325 | 6.277 |
|  | 6 months | 14 | 6.20 | 0.763 | 6.43 | 5.764 | 6.645 | 14 | 6.10 | 0.811 | 6.27 | 5.632 | 6.568 |
| Change in Mini-AQLQ Subscore Symptoms | 3 months | 14 | 5.60 | 0.891 | 5.80 | 5.086 | 6.114 | 27 | 5.61 | 1.290 | 6.00 | 5.104 | 6.125 |
|  | 6 months | 14 | 5.99 | 0.798 | 6.20 | 5.525 | 6.446 | 14 | 5.99 | 1.254 | 6.30 | 5.262 | 6.710 |
| Change in Mini-AQLQ Subscore Emotions | 3 months | 14 | 5.79 | 1.195 | 5.67 | 5.095 | 6.476 | 27 | 5.98 | 1.205 | 6.33 | 5.499 | 6.452 |
|  | 6 months | 14 | 6.24 | 0.982 | 6.83 | 5.671 | 6.805 | 14 | 6.07 | 0.908 | 6.17 | 5.547 | 6.595 |
| Change in Mini-AQLQ Subscore Environment | 3 months | 14 | 5.67 | 1.536 | 6.33 | 4.780 | 6.553 | 27 | 5.49 | 1.412 | 5.67 | 4.935 | 6.053 |
|  | 6 months | 14 | 6.31 | 0.929 | 6.67 | 5.773 | 6.846 | 14 | 5.98 | 1.362 | 6.67 | 5.190 | 6.762 |
| Change in Mini-AQLQ Subscore Activity | 3 months | 14 | 6.25 | 0.778 | 6.50 | 5.801 | 6.699 | 27 | 6.14 | 1.336 | 6.75 | 5.610 | 6.667 |
|  | 6 months | 14 | 6.38 | 0.758 | 6.63 | 5.937 | 6.813 | 14 | 6.36 | 0.648 | 6.50 | 5.983 | 6.731 |

*Complete cases only – no imputed values.*

Using random effects models the ICC was derived from the results. Values of ICC were estimated to be 0.0705 at baseline, 0.0562 at 3 months and 0.0943 at 6 months for the mAQLQ. These are consistent with the often assumed value of 0.05 for outcomes in cluster randomised trials.
