# Supplementary material for: Achieving Good Outcomes for Asthma Living (GOAL): mixed methods feasibility and pilot cluster randomised controlled trial of a practical intervention for eliciting, setting and achieving goals for adults with asthma
Source: Trials. 2016 Dec 8;17:584. doi: 10.1186/s13063-016-1684-7 (PMC5146838; doi:10.1186/s13063-016-1684-7)
Supplement: Additional file 3: — Provides a breakdown of health service resource use costs in the intervention and control groups. (DOCX 53 kb) [file 13063_2016_1684_MOESM3_ESM.docx]

**Additional File 3**

Mean Cost and QALYs

|  | **Intervention group** | | **Control group** | | **Difference** |
| --- | --- | --- | --- | --- | --- |
|  | **N** | **Mean (SD)** | **N** | **Mean (SD)** |  |
| **Intervention costs** | 16 | 19.30 (7.53) | 15 | 0.00 |  |
| **Total Primary care** | 16 | 43.31 (73.63) | 15 | 20.20 (15.96) |  |
| General Practitioner | 16 | 24.75 (71.66) | 15 | 4.80 (12.67) |  |
| Practice nurse | 16 | 18.56 (12.52) | 15 | 15.40 (9.11) |  |
| Out of hours | 16 | 0.00 | 15 | 0.00 |  |
| Specialist respiratory nurse | 16 | 0.00 | 15 | 0.00 |  |
| **Total Secondary Care** | 16 | 0.00 | 15 | 0.00 |  |
| Inpatient stays | 16 | 0.00 | 15 | 0.00 |  |
| Accident & Emergency | 16 | 0.00 | 15 | 0.00 |  |
| Outpatient visit | 16 | 0.00 | 15 | 0.00 |  |
| **Asthma medication** | 16 | 115.31 (80.05) | 15 | 95.77 (113.84) |  |
| **TOTAL COST** | 16 | 177.92 (119.30) | 15 | 115.97 (121.15) |  |
| **TOTAL COSTS (adjusted for baseline differences)^**^** | 16 | 158.68 |  | 136.50 | p-value GLM: 0.52^***^ |
|  |  |  |  |  |  |
| EQ5D - baseline | 18 | 0.827 (0.193) | 30 | 0.847 (0.230) |  |
| EQ5D – 3 months | 15 | 0.813 (0.278) | 27 | 0.892 (0.165) |  |
| EQ5D – 6 months | 15 | 0.801 (0.306) | 15 | 0.850 (0.271) |  |
| **Generic QALYs^*^** | 15 | 0.409 (0.122) | 15 | 0.448 (0.099) |  |
| **Generic QALYs adjusted for baseline differences^**^** |  | 0.415 |  | 0.442 | p-value GLM: 0.50^***^ |

*Last Value Carried Forward for 3 respondents with 1 missing value; ^**^Adjusted for baseline by using Generalised Linear Modelling (GLM) ^***^ p-value based on bootstrapped standard errors.
